# The interaction between DNA methylation and tumor immune microenvironment: from the laboratory to clinical applications

**DOI:** 10.1186/s13148-024-01633-x

**Published:** 2024-02-08

**Authors:** Daoqi Zhu, Siying Zeng, Chao Su, Jingjun Li, Yiwen Xuan, Yongkai Lin, Enwu Xu, Qin Fan

**Affiliations:** 1 School of Traditional Chinese Medicine, Southern Medical University, No. 1023 Shatai North Road, Guangzhou, 510515 China; 2 Department of Thoracic Surgery, General Hospital of Southern Theater Command, PLA, No.111 Liuhua Road, Guangzhou, 510010 China; 3 Nanfang Hospital, Southern Medical University, Guangzhou, 510515 China; 4 Department of Endocrinology, The First Affiliated Hospital, Traditional Chinese Medicine University of Guangzhou, Guangzhou, 510405 China

**Keywords:** DNA methylation, Tumor immune microenvironment, Epigenetic regulation, Immune cell function, Immunotherapy challenges

## Abstract

DNA methylation is a pivotal epigenetic modification that affects gene expression. Tumor immune microenvironment (TIME) comprises diverse immune cells and stromal components, creating a complex landscape that can either promote or inhibit tumor progression. In the TIME, DNA methylation has been shown to play a critical role in influencing immune cell function and tumor immune evasion. DNA methylation regulates immune cell differentiation, immune responses, and TIME composition Targeting DNA methylation in TIME offers various potential avenues for enhancing immune cytotoxicity and reducing immunosuppression. Recent studies have demonstrated that modification of DNA methylation patterns can promote immune cell infiltration and function. However, challenges persist in understanding the precise mechanisms underlying DNA methylation in the TIME, developing selective epigenetic therapies, and effectively integrating these therapies with other antitumor strategies. In conclusion, DNA methylation of both tumor cells and immune cells interacts with the TIME, and thus affects clinical efficacy. The regulation of DNA methylation within the TIME holds significant promise for the advancement of tumor immunotherapy. Addressing these challenges is crucial for harnessing the full potential of epigenetic interventions to enhance antitumor immune responses and improve patient outcomes.

## Introduction

Epigenetics refers to the scientific study of reversible alterations in gene expression and function that result in heritable phenotypic changes, while preserving the underlying DNA sequence. Histone modifications, non-coding RNA regulation, and alterations in DNA methylation are important epigenetic mechanisms. One of the oldest and most important epigenetic alterations is DNA methylation [1], assumes a critical role in embryonic development, X-chromosome inactivation, gene imprinting, and transposon activity control.

**Fig. 1:**
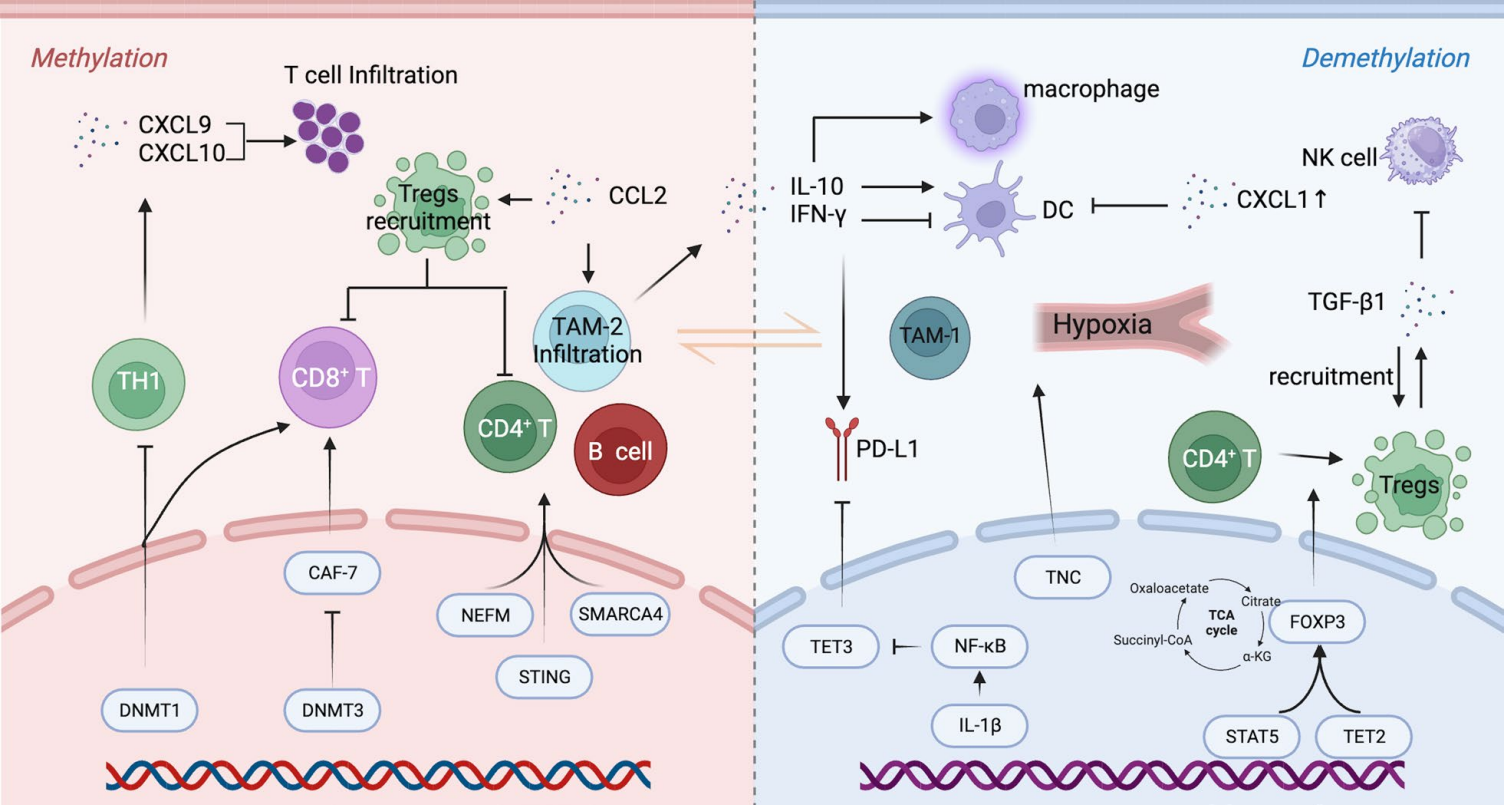
The perspectives and relevant mechanisms of this review. Methylation-driven remodeling is intricately associated with the maintenance or alteration of the TME, particularly the TIME. The methylation of tumor suppressor genes (left) or the demethylation of proto-oncogenes (right) is closely linked to the initiation and progression of cancer. The TIME is generally classified into immune-exempt “cold tumors” and inflamed “hot tumors” phenotypes. DNA methylation reprogramming exerts multifaceted regulation on the TIME through its influence on the infiltration, differentiation, secretion of various tumor immune cells (Tregs, B cells, macrophages), vascular genesis (hypoxic microenvironment), metabolism (lipids, mitochondria), and other aspects

Furthermore, DNA methylation reprogramming, prominently observed in various diseases, particularly malignancies and immune-related disorders, is closely associated with the tumor microenvironment (TME), including the tumor immune microenvironment (TIME). Recent research has highlighted the intricate relationship between methylation remodeling and the TME, particularly the TIME. A profound understanding of the inherent mechanisms governing the interplay between DNA methylation and the TIME may reveal a novel avenue for cancer combination immunotherapy, achieved by targeted manipulation of DNA methylation [2, 3]. Figure 1 illustrates the perspectives and relevant mechanisms used in this review. This article primarily focuses on a comprehensive discourse concerning the interactions between DNA methylation regulatory networks and the TIME, along with potential collaborative therapeutic approaches.

## Molecular level of the TIME and DNA methylation reprogramming

DNA methylation is an epigenetic process involving the addition of methyl groups to cytosine residues. This modification occurs at the C5 position of cytosine and is catalyzed by enzymes called DNA methyltransferases (DNMT), leading to the formation of 5-methylcytosine (5mC). In vivo, DNA methylation dynamically regulates gene modification and maintains equilibrium through methylation, recognition, and removal. Three active DNMTs have been identified in mammals and are denoted as DNMT1, DNMT3a, and DNMT3b [4–6]. DNA demethylation primarily involves mediation by the ten-eleven translocation (TET) family of enzymes. In the active demethylation pathway, the dioxygenase domain catalyzes the hydroxylation of 5mC to generate 5-hydroxymethylcytosine, which is further converted to 5-formylcytosine and 5-carboxylcytosine. Subsequent processes involve the dilution of DNA methylation through replication or the removal of bases through thymine DNA glycosylase-dependent base excision repair, resulting in changes in DNA methylation. TET family dioxygenases and DNA demethylation exhibit pleiotropic biological effects in both stem cells and cancer cells [7] and play significant roles in immune cell differentiation and maturation [8]. Three active mammalian TET enzymes have been identified to date, TET1, TET2, and TET3 respectively [9–13].

The TME is an intricate assemblage of stromal cells, tumor cells, and the extracellular matrix (ECM), encompassing immune cells, inflammatory cells from the bone marrow, fibroblasts, blood vessels, and various signaling molecules. Notably, the TIME is a vital component of the TME, comprising T cells, B cells, macrophages, dendritic cells, tumor-associated fibroblasts, neutrophils, and secreted cytokines, collectively comprising various infiltrating immune cells [14]. The composition and abundance of stromal cell types dictate TIME characteristics, further influencing tumor progression and immune responses [2, 3, 15]. The heterogeneity of the TIME results in significant differences in tumor progression between individuals. Generally, the TIME can be categorized as either immune-inflamed (“hot tumors”) or immune-excluded (“cold tumors”) [2, 3, 16].

### DNA methylation and the TIME modulating

The mechanisms by which DNA methylation influences TIME can be summarized as methylation of tumor suppressor genes, which subsequently execute immune defense or immune cytotoxic functions, and demethylation of oncogenes, which perform immune suppression or immune tolerance functions. Table 1 presents the statuses of some of the relevant genes reported to date. Reversing methylation/demethylation may offer potential novel epigenetic therapeutic interventions targeting the TIME.

**Table 1:** Gene List of DNA Methylation/Demethylation and related TIME alter

Methylation/demethylation	Gene	Immunomodulatory effect	Prognosis	Tumor	PMID
DNA methylation	NEFM	Positively correlated to macrophage infiltration	Poor prognosis	Breast cancer; Glioma	3400120832450002
	SMARCA4	Negative correlation with the infiltration of CD8+ T cells	Better prognosis	Pan-cancer	34675941
	STING1	Negative correlation with the infiltration of CD8+ T cells	Poor prognosis	Lung cancer	35978045
	CYTIP	Improving anti-PD-1 immunotherapy	Poor prognosis	NSCLC	32879421
	TNFSF8	Improving anti-PD-1 immunotherapy	Poor prognosis	NSCLC	32879421
	GPC2	Positively correlated with infiltrated T cells, auxiliary T cells, Tcm, Th17 cells and Th2 cells	Poor prognosis	Bladder cancer, Breast cancer	35345673
	TNFRSF9	positively correlated with immune cell infiltrates and an interferon-γ signature	Poor prognosis	Melanoma	3202068
	RNF135	Positively correlated with B cells, CD4 T cells, macrophages and dendritic cells	Poor prognosis	Hepatocellular carcinoma	35145901
DNA demethylation	FOXP3	Regulate the stability and inhibition function of Tregs, and directly regulate the amplification and function of T cells	Poor prognosis	NSCLC	27000869
	FOXP1	Regulate macrophage hypoxia	Better prognosis	NSCLC	30100403
	Tenascin-C	the morphology and function of TAM	Poor prognosis	Glioma	32845507
	DDOST	Negative correlation with the infiltration of B cells and CD4+ T cells	Poor prognosis	Glioma	35812432
	ERBB2	suppress induction and proliferation of effector T cells	Poor prognosis	Pan-cancer	36172165
	APOBEC3H	CD8+ T cell immune infiltration and activation	Better prognosis	Squamous cell carcinoma of the head and neck	32775421

There is a growing consensus that the DNA methylation of tumor suppressor genes (immune defense genes) is closely associated with cancer development. In a study conducted by Li et al. [17], it was cancer patients with high levels of neurofilament medium polypeptides (NEFM) exhibited improved overall survival (OS) and recurrence-free survival (RFS). Conversely, DNA methylation in NEFM is associated with worse OS, potentially owing to reduced NEFM expression. Upon further examination, it was discovered that there was a noteworthy inverse relationship between NEFM DNA methylation levels and the infiltration levels of CD4+ T cells, CD8+ T cells, B cells, macrophages, dendritic cells, and neutrophils in tumors, with a positive correlation observed with macrophage infiltration. These results suggest that NEFM DNA methylation leads to a poorer prognosis in patients by modulating the breast TIME. One possible mechanism for this phenomenon is that the absence of NEFM in breast tumor cells causes intermediate filaments to become unstable, which in turn causes cytoskeletal disarray and dynamic cell deformation. This, in turn, facilitates tumor EMT and interferes with immune microenvironment signaling, increasing the motility of tumor cells and their capacity to colonize nearby tissues [18].

Zinc finger and SCAN domain-containing protein 18 (ZSCAN18) is a crucial member of a family of transcription factors associated with the cell cycle and glycolysis signaling pathways. It can bind to the promoter of tumor protein 53-induced nuclear protein 2 (TP53INP2) and modulate the anti-tumor response [19]. An additional study by Wang et al. discovered that whereas relatively high expression of ZSCAN18 is linked to a favorable prognosis, DNA methylation-modified ZSCAN18 is underexpressed in breast tumors. Breast tumor tissues have higher levels of ZSCAN18 DNA methylation than normal tissues. Numerous genes linked to the Wnt/β-catenin and glycolysis signaling pathways can be inhibited by ZSCAN18 upregulation. ZSCAN18 expression negatively correlated with infiltrating B cells and DC, whereas ZSCAN18 DNA methylation positively correlated with activated B cells, CD8+ and CD4+ T cells, macrophages, neutrophils, and DC. According to the study, DNA methylation modifications significantly influence the TIME through transcriptional regulation and the glycolysis signaling pathway.

STING, an essential tumor suppressor gene and vital regulator of tumor immunity, has been shown to play an important role in tumor suppression and immune control [20]. Recently, Lin et al. [21] found that both mRNA and protein expression of STING are reduced in adenocarcinoma (LUAD), and that LUAD patients with low STING expression have a poorer prognosis, which may be due to the hypermethylation of STING. Similar investigations have demonstrated a strong relationship between STING methylation and the TIME. A549 and H1975 are two LUAD cell lines whose proliferation and metastasis are inhibited by TET2 overexpression. However, these cells proliferate, migrate, and invade more readily when TET2 is knocked down. Mechanistically, LUAD carcinogenesis and metastasis were significantly influenced by the TET2-mediated DNA methylation balance in STING. [22] In order to activate STING and produce Type I IFN, which in turn restores the CD8+ T cell-dependent immune response in tumor-bearing mice, Falahat R employed DNA methylation inhibitors to reverse the methylation silent of STING in mouse melanoma cells [23]. According to a previously described study, one potential new avenue for the therapy of certain malignancies is to reverse the methylation status of STING via epigenetic reprogramming, which would modify the TIME.

### DNA demethylation and the TIME modulating

Even though certain immunological tolerance-related genes or proto-oncogenes that are demethylated have been strongly linked to tumor formation, as well as the intrinsic roles of these genes, they have received relatively little research attention [24, 25]. Initially considered a Tregs-specific expression molecule, Forkhead box protein 3 (FOXP3) was believed to be involved in regulating immune-suppressive functions. Recent studies have revealed FOXP3 expression in various cancers, including gastric [26], pancreatic [27], liver [28], and breast [29] cancer [29]. Although its functional role may vary [30–32], numerous cancer studies have suggested that FOXP3 is highly expressed in tumor cells or T-cells, and that this high expression is associated with FOXP3 demethylation.

Ke et al. [33] observed that compared to healthy individuals, individuals with non-small cell lung cancer (NSCLC) have an increased prevalence of CD4+ Treg cells in the bloodstream. Moreover, individuals with NSCLC were found to have demethylation at eight CpG sites in the *foxp3* promoter, with methylation levels showing a negative correlation with the proportion of CD4+ CD25+ Foxp3+ T cells. In vitro studies showed that tumor cells impacted the function of CD4+ Tregs, leading to the secretion of IL-10 and TGF-β1. Furthermore, a decrease in DNA methyltransferase activity was observed in CD4+ T cells, leading to demethylation of eight CpG sites in the *foxp3* promoter. These findings indicate that the increased expression of FOXP3 in CD4+ T cells may be a result of demethylation of the promoter region. Tregs exhibit strong immunosuppressive effects and significantly inhibit the proliferation of naïve CD4+ T cells. This study confirmed that tumor cells in patients with NSCLC downregulate immune responses promoted tumor progression by affecting *foxp3* promoter demethylation in T cells. In line with the demethylation of T-cell FOXP3, Schultze et al. [32] examined the correlation between the demethylation of FOXP3 in tumor cells and the TIME. The results showed that the average frequency of cells with demethylated FOXP3 in normal tissues was significantly lower than that in tumor tissues from both patients with colorectal cancer (CRC) and intrahepatic cholangiocarcinoma (ICC) rats. This suggests that FOXP3, a Treg biomarker, may play an intriguing role in immune evasion induced by tumor cells.

Thrombospondin-2 (THBS2) is a glycoprotein of the extracellular matrix that effectively prevents tumor development and angiogenesis. The expression of THBS2 is dramatically elevated in colon adenocarcinoma (COAD), as shown by Liu et al. [33]. It is negatively correlated with DNA replication, repair, and the cell cycle, and favorably correlated with angiogenesis and epithelial-mesenchymal transition. The expression of THBS2 has a favorable correlation with microsatellite instability and a substantial relationship with the progression-free interval in COAD. THBS2 methylation levels in COAD tissues were markedly lower than those in healthy tissues. Nuclear translocation of HIF1 is greatly increased by the high exogenous expression of THBS2 in CT26 cells, which enhances intracellular lactate metabolism. Additional studies conducted both in vitro and in vivo suggest that lactate generated by tumor cells stimulates macrophage M2 polarization, which in turn prevents T cell proliferation and destruction. By mediating DNA methylation modifications, THBS2 functions as a mediator between the tumor extracellular matrix and immune infiltration, thereby influencing biological processes, including immune cell infiltration, immune regulation, cell death, migration, epithelial-mesenchymal transition, and angiogenesis [34].

Tenascin-C (TNC), which was first discovered in the 1980s, is a multidomain extracellular matrix glycoprotein that is highly expressed during multicellular organisms [35]. TNC levels are generally undetectable in most adult tissues, likely because of epigenetic silencing during embryonic development. Sustained TNC expression is associated with chronic inflammation and many malignant tumors, including prostate cancer, glioblastoma, and nasopharyngeal carcinoma (NPC) [25, 26, 36]. Through interactions with their receptor integrins and numerous other binding components, TNC trigger environmental and cell type-dependent functions to regulate cell adhesion, migration, proliferation, and angiogenesis. Additionally, it plays a role in tumor epithelial-mesenchymal transition (EMT) and TME modulation [37]. TNC, an endogenous TLR4 activator, enhances inflammatory responses by increasing the production of pro-inflammatory cytokines in innate immune cells such as macrophages and microglia. Furthermore, TNC promotes macrophage differentiation and polarization toward an M1-like phenotype, whereas TNC exhibits immunosuppressive functions in T cells. In glioblastomas, TNC is expressed in the tumor and stromal cells, and its high expression is associated with tumor progression and poor prognosis. In addition to promoting glioblastoma invasion and angiogenesis, TNC affect the morphology and function of tumor-associated microglia/macrophages, suggesting that TNC contribute to glioblastoma progression by influencing EMT and TIME [36].

In a recent study conducted by our team [25], we found that TNC underwent significant demethylation in radioresistant NPC cell strains with high TNC expression. The traditional Chinese medicine Shengmai Yin is radiosensitive and partially restores the demethylated state of radioresistant NPC cell strains, suggesting that TNC demethylation is involved in the remodeling of the NPC radiation microenvironment. During this process, the radiation microenvironment may directly or indirectly interact with the immune microenvironment, thereby collectively influencing the TME.

In addition to their direct effects on the immune-related genes, DNA methylation and demethylation may indirectly shape the TIME through various mechanisms, including the EMT [38–40]. The delicate equilibrium between DNA methylation and demethylation [41] is crucial for the normal functioning of cells and development of mammals. DNMTs, TETs, and related enzymes collectively regulate this physiological balance and have significant implications for the interactions between cancerous tumors and the immune system. Disruption of this balance in the TME may be an important potential avenue for DNA methylation-related tumor immunotherapy or adjunctive therapy.

## DNA methylation remodeling and the cellular level of tumor immunotherapy

Reversing this process, which involves the remodeling of DNA methylation and disruption of the TIME, has become an important avenue in tumor immunotherapy. The distribution and proportions of various functionally distinct immune cells within the TIME determine whether a tumor is “hot” or “cold” [3]. DNA methylation is strongly linked to the maturation, polarization, differentiation, and function of immune cells, and contributes to tumor evasion from the immune system [2, 3]. The following sections elaborate on the current research status of enhancing immune cytotoxicity and reducing immune escape/suppression through DNA methylation remodeling to regulate the TIME and immunotherapy.

### Regulating the TIME by enhancing immune cytotoxicity through DNA methylation remodeling

The epigenetic regulation of CD8+ T cells plays a crucial role in acquiring and maintaining immune cytotoxicity as well as in mounting rapid and robust responses to antigen re-challenges. CD8+ T-cells of various cells exhibit dynamic methylation patterns at various stages of differentiation For example, in response to severe and frequent antigen stimulation, transcription factors (TFs) associated with effector T cells bind to particular demethylated sites in genes such as IFNG and GZMB, resulting in effector phenotypic differentiation [42]. Since that increases the methylation of the promoter of T cell-specific transcription factor 7 (TCF7), DNMT3A is required for CD8+ effector T-cell development [43]. TCF7 encodes TCF1, a transcription factor that is upregulated in naïve T cells and central memory T cells but downregulated in effector memory T cells. TCF7 silencing impairs stem cell-like T cell renewal and central memory CD8+ T cell memory response [44]. Despite the complexity of the epigenetic regulation of CD8+ T cells in tumor formation [45], various studies have shown that suppressing DNMTs may successfully boost the antitumor effects of CD8+ effector T cells and alter the TIME [46].

Tumor-infiltrating lymphocytes (TILs), particularly CD8+ TILs, are closely linked to the TME immune landscape. These markers serve as valuable prognostic indicators of responsiveness to immunotherapy and patient survival. In a study by Zou et al. [47] that focused on CRC, three CD8+ T cell-specific differentially methylated regions were identified, which enabled the establishment of a CD8+ MeTIL feature score. These findings revealed that lower CD8+ MeTIL scores, indicating enriched CD8+ TILs, correlated with favorable prognoses in patients with CRC. Ovarian cancer (OCs), a notably lethal gynecological malignancy, is a subset of diseases with a limited response to prevailing immunotherapies. This phenomenon can be attributed to modulation of the TIME and inadequate recruitment and activation of immune cells, which are essential for the elimination of cancer cells. Gomez et al. [48] observed that DNA methyltransferase inhibitors (DNMTis) activate the immune-suppressed TME in OC. This activation mechanism involves the restructuring of methylation patterns, subsequently leading to the recruitment and activation of more CD8+ T cells. Moufarrij et al. [49] found that combining DNMTis and HDAC6 inhibitors enhanced the Type I interferon response, leading to an increased production of cytokines, chemokines, and MHC I antigen presentation complex components in OC cells. This alteration in the TIME is characterized by a rise in tumor-killing cells including interferon (IFN)γ+ CD8, NK, and NKT-cells, accompanied by a reduction in MDSCs and PD-1hi CD4 T-cells, ultimately reversing the immune-suppressive TME.

CD4+ T-cells, under the supervision of several TFs, differentiate into various T-helper cell subsets. Epigenetic processes play integral roles in the development and function of Th1 and Th2 cells. The role of Th1 cells in immunological cytotoxicity in the TIME is discussed here. Travers et al.[13] illustrated that medicine with the combination of 5-azacytidine (5AZA-C) and α-difluoromethylornithine (DFMO) can induce the recruiting process of activated CD8+ T-cells, IFNγ+ CD4+ T-cells (Th1), and NK cells, while significantly reducing immunosuppressive cells like M2-polarized macrophages and increasing tumor-killing M1 macrophages, in the TIME of patients with OC. Li et al. [50] proposed that epigenetic modifications might lead to the abnormal regulation of angiogenesis and the TME. They found that the prognosis of patients with NSCLC treated with chemotherapy combined with bevacizumab differed based on their DNA methylation patterns. Significant enrichment of differentially methylated regions in genes related to the VEGFA-VEGFR2 signaling pathway, neutrophil-mediated immunity, neutrophil degranulation, and other biological processes was observed. Peng et al. [51] discovered that DNMT1 mediates DNA methylation in OC, decreasing the synthesis of Th1-secreted chemokines CXCL9 and CXCL10, and increasing effector T-cell infiltration. Furthermore, the expression levels of tumor DNMT1 were negatively associated with infiltrating CD8+ T cells and patient prognosis. According to this study, targeted epigenetic remodeling alters T-cell distribution and may enhance the clinical efficacy of cancer therapies. This also suggests that the epigenetic suppression of Th1-type chemokines represents a distinct mechanism of cancer immune evasion.

### Regulating the TIME by reducing immune suppression through DNA methylation remodeling

Compared to antitumor immune cells, the TIME harbors a greater proportion of immune-inhibitory cells that are associated with tumor immune evasion. These immune-inhibitory cells progressively develop immune escape mechanisms during tumor development. Major players in this category include Tregs, MDSC, and TAM-2s [52]. CD4+ CD25+ T cells are a subset of suppressive T cells that play a dominant role in downregulating or inhibiting the induction and proliferation of effector T cells and mediating peripheral immune tolerance [53]. Within the TIME, Tregs are considered the primary immune suppressive factors, and they employ various mechanisms to dampen immune responses [54–56]. One of the key challenges in Treg-based therapy is finding effective strategies to inhibit Tregs while activating TILs without compromising the body's immune functions [57, 58].

The role of Foxp3 in the development and suppression of Tregs makes it a prime target for epigenetic therapies that target Tregs. Sabir et al. [59] discovered a strong negative association between the efficacy of imatinib therapy in patients with late-stage and optimal-response chronic myeloid leukemia, Treg demethylation percentage, and the Foxp3 Treg-specific demethylation region (TSDR). According to Ma et al. [60], increased STAT5 expression in CRC CD4+ T cells can attract additional TET2 to FOXP3-TSDR, resulting in elevated FOXP3 expression via DNA demethylation. This highlights the mechanism underlying low methylation of FOXP3-TSDR in tumor-infiltrating CD4+ T cells in patients with CRC. Other studies on FOXP3/TSDR have provided similar insights [61, 62]. Changes in cell-permeable ketoglutarate (KG) modify the DNA methylation profile of initial CD4+ T-cells stimulated under Treg polarization conditions, considerably decreasing FOXP3+ Treg differentiation and enhancing inflammatory cytokine production, according to Matias et al. [63]. This research suggests that altering αKG, mitochondrial metabolism, and lipid homeostasis represents a novel approach to inhibiting Tregs and enhanceing immune therapy in the TIME.

Within the TIME, MDSCs, which originate from immature bone marrow precursors, play a crucial role in facilitating tumor immune evasion [64, 65]. They activate the immunosuppressive activity of Tregs by modulating the production of interleukin (IL)-10 and IFN- and enhance the conversion of initial CD4+ T cells into Tregs by secreting retinoic acid and TGF- [66]. Furthermore, MDSCs can directly affect the activities of CD8+ T cells and NK cells in the TIME via cell–cell interactions and soluble factor release. Furthermore, MDSCs can establish T cell tolerance by expressing inhibitory receptors such as PD-L1 and cytotoxic T lymphocyte-associated antigen 4 (CTLA4), MDSCs can establish T-cell tolerance [67]. Numerous studies have found that DNA methylation influences MDSC development, maturation, and recruitment to the TME [68, 69]. Smith et al. [70] reported that the DNMTi decitabine (DAC) reduces the accumulation of MDSCs in mice with CRC, facilitating the activation of antigen-specific cytotoxic T lymphocytes. This mechanism may be attributed to the modulation of TNF promoter demethylation by DAC, which decreases DNMT expression and subsequently blocks RIP1-dependent necrotic target methylation, enhancing cell death and reducing MDSC accumulation. Guadecitabine was demonstrated in another trial by Luker et al. [71] to considerably reduce tumor burden by blocking excessive bone marrow proliferation and systemic MDSC accumulation in a T cell-dependent manner. The remaining MDSCs transitioned to the antigen-presenting phenotype. The findings of this study suggest that guadecitabine has the potential to enhance the therapeutic efficacy of adoptive transfer of APLs, leading to a decrease in tumor growth and an improvement in overall survival rates. Collectively, these findings suggest that epigenetic remodeling could serve as an effective strategy to inhibit MDSCs and regulate the TIME.

TAMs are derived from monocytes in the blood and migrate to solid tumor tissues. They are divided into two separate subgroups, M1 and M2, each with dramatically different roles [72]. TAM-1s are primarily activated by factors such as IFN-γ, IL-2, and TNF-γ. They play a role in polarizing Th1 responses, recruiting cytotoxic T lymphocytes (CTLs), and promoting antitumor immune responses. In contrast, TAM-2s are stimulated by IL-4 and IL-13, leading to the development of an immunosuppressive TME. They secrete factors like TGF-β, suppress NK cell activity, and upregulate the expression of PD-L1 and CTLA4. This ultimately hinders the infiltration of effector T cells by blocking immune checkpoints [73]. Moreover, TAM-2s can dampen immune responses by producing anti-inflammatory cytokines such as TGF-β, IL-10, IL-13, and IL-4, which inhibit CTL functions and upregulate Tregs, thereby weakening the immune response [74, 75]. Differentiation of TAMs and their transition between the M1 and M2 phenotypes are also subject to epigenetic regulation [76]. Travers et al. [13] previously reported that combination therapy with 5AZA-C and DFMO in patients with OC resulted in a substantial decrease in immunosuppressive cells, such as M2-polarized macrophages, and an increase in TAM-1s. This study suggests that altering macrophage polarization in the TME and recruiting TAM-1s can extend the survival of patients with OC. Furthermore, research by Zhang et al. [77] revealed that during the development of pancreatic ductal adenocarcinoma (PDA), tumor cells induce selective Nqo-1 DNA methylation in TAM-1s through direct cell-to-cell contact mediated by GARP and integrins αV/β8. This leads to suppression of the glycolytic state in TAM-1s, ultimately reprogramming them to become TAM-2s. These findings suggested that PDA cells can reprogram TAM-1s through DNA methylation-mediated mechanisms of metabolism and function. Epigenetic reprogramming, through either epigenetic modifications or drug interventions, has the potential to reverse the transition from M2 to M1, thereby offering a novel approach for cancer therapy.

Currently, immunotherapies that rely on reprogramming DNA methylation, aimed at either enhancing immune cytotoxicity or reducing immune tolerance, remain limited (Table 2). To date, research has primarily focused on non-solid tumors and the demethylation of tumor suppressor genes (Table 3). Therapies that actually target oncogene methylation remain relatively unknown, and progress in this area depends on further in-depth research into the relevant mechanisms. There will be significant advancements in treatments targeting oncogene methylation in the near future.

**Table 2:** The list of combining demethylating drugs and immunotherapy under clinical trials

Demethylating drugs	Immunotherapy	Cancer types	Study Start	Study location	Phase	ClinicalTrials.gov ID
Decitabine	Pembrolizumab	HER2-negative breast cancer	2017	United States	Phase 2	NCT02957968
Decitabine	Pembrolizumab	relapsed, refractory or progressive non-primary CNS solid tumors and lymphomas	2018	United States	Early Phase 1	NCT03445858
Decitabine	Pembrolizumab	Non-small cell lung cancer	2018	United States	Phase 1 Phase 2	NCT03233724
Decitabine	Nivolumab	Unresectable or Metastatic Mucosal Melanoma	2022	United States	Phase 1 Phase 2	NCT05089370
Decitabine	Tirelizumab	Advanced Esophageal Squamous Cell Carcinoma	2023	China	Phase 2	NCT05638984
Decitabine	Anti-PD-1 antibody	Relapsed or refractory malignancies	2016	China	Phase 1 Phase 2	NCT02961101
Decitabine	MBG453; PDR001	Advanced/metastatic solid tumors	2015	United States	Phase 1 Phase 2	NCT02608268
ASTX727	Nivolumab	Relapsed or Refractory Diffuse Large B-Cell Lymphoma	2022	United States	Phase 1	NCT05272384
ASTX727	Durvalumab	recurrent or metastatic squamous cell carcinoma of the head and neck	2017	United States	Phase 1 Phase 2	NCT03019003
Azacitidine	Pembrolizumab	Relapsed/Refractory Hodgkin's Lymphoma	2022	United States	Phase 2	NCT05355051
Azacitidine	Pembrolizumab	Metastatic Melanoma	2017	United States	Phase 2	NCT02816021
Azacitidine	Pembrolizumab	Chemo-refractory Metastatic Colorectal Cancer	2015	United States	Phase 2	NCT02260440
Azacitidine	Pembrolizumab	Pancreatic Cancer	2017	United States	Phase 2	NCT03264404
Azacitidine	Pembrolizumab	Advanced Solid Tumors	2017	United States	Phase 1 Phase 2	NCT02959437
Azacitidine	Nivolumab	Metastatic Non-Small Cell Lung Cancer	2013	United States	Phase 2	NCT01928576
5-Azacytidine	Nivolumab	Resectable HPV-Associated Head and Neck Squamous Cell Cancer	2023	United States	Phase 1	NCT05317000
CC-486	Nivolumab	Hodgkin Lymphoma Refractory	2022	United States	Phase 1	NCT05162976
Guadecitabine	Durvalumab	Advanced kidney cancer	2017	United States	Phase 1 Phase 2	NCT03308396
Guadecitabine	Atezolizumab	Refractory or Resistant Urothelial Carcinoma	2017	United States	Phase 2	NCT03179943

**Table 3:** The list of DNA methyltransferase inhibitors and related TIME or tumor alter

DNMTi	Function	Combined use	Tumor	PMID
Decitabine	Increased expression of cancer-testis antigens	–	Ovarian cancer	26098711
	Tumor cell lysis by CTL	PD-L1/PD-1 blocker	Rhabdomyosarcoma	32528824
	Increased memory T cell infiltration and up-regulation CTLA-4 and FOXP3	ipilimumab	Melatoma	36706355
	CTL-mediated tumor cell killing	IFN-γ	Neuroblastoma	21626030
	PD-1 blocking	Pembrolizumab	Leukemia	35017151
	Increased B lymphocytes	CAR-T	Lymphoma	36059523
	Enhance the secretion of ifn-γ and mage-a3 antigen-sive t cells	–	Esophageal squamous cell carcinoma	30797153
	Upregulation the genes involved in congenital and adaptive immunity and PD-L1	Nivolumab	NSCLC	34140403
	Reverse the depletion of CD8+ TIL and improve T cell response	Nivolumab	gastric cancer	35024441
Azacytidine	Inducing apoptosis of p53-dependent cells	Nivolumab	squamous cell carcinoma of the head and neck	28916527
	Down-regulate B-cell lymphoma 2	venetoclax	Acute myelogenous leukemia	32054729
	Reduce the suppressive function of Tregs	–	Myelodysplastic syndrome	23242597
	Upregulate the genes involved in congenital and adaptive immunity and PD-L1	Nivolumab	NSCLC	29195073
	Increased plasma HMGB1 expression	–	Osteosarcoma	29097772
Zebularine	Promote the infiltration of CD8 T cells and NK cells	–	Myeloma	32394351
Hydralazine	Upregulate HLA-1 antigen expression and antigen-specific CTL response	Valproic acid	Cervical cancer	17192185
	Induce ICD and CTL infiltration	bortezomib	Pan-cancer	36031455
EGCG	Reduce TAM to inhibit tumor growth	Genistein	Pan-cancer	31877341

## Prospects and challenges

Regulation of DNA methylation and immunotherapy are becoming increasingly prominent research fields. Convincing evidence suggests that epigenetic modifications influence the interactions between cancer, immune cells, and stromal cells, ultimately regulating the state of the TIME. Therefore, alterations in DNA methylation have the potential to open new avenues of cancer therapy. Although drugs targeting DNA methylation modifications have been explored for clinical applications, they are still in relatively early stages of development and will face significant challenges in the future.

Further development and maturation are imperative for basic research. There remains a need for an in-depth investigation into the mechanisms linking DNA methylation and the TIME. The depth of our understanding of this relationship directly affects the precision of practical applications. Key aspects that require further elucidation include the mechanisms governing the selective action of methylation enzymes on target cells and the intricate association between oncogene demethylation and tumorigenesis. This will depend on the emergence of new epigenetic technologies. For instance, novel detection technologies like next-generation sequencing enable high-throughput methylation site detection and accurate identification of various DNA/RNA methylation patterns, such as m5c, m6a, m7g [78–80]; these technologies also function as tumor biomarkers to inform precise disease subtyping and customized treatment [81]; concerning modification technologies, CRISPR-based epigenomic editing tools precisely manipulate DNA methylation at specific genomic sites, modifying methylation at selected biomarker locations to provide more targeted therapy[82]; concerning analysis technologies, these include the integrated analysis of multi-omics data and the simulation of treatment plans and outcomes using machine learning [83].

Second, the use of DNMT-based drugs poses significant challenges. DNA methylation modifications are pervasive, occurring in both normal and neoplastic cells, and these drugs exhibit varying degrees of function depending on the cell type. The conundrum lies in the precise targeting of tumor cells by broad-spectrum DNMTis during therapeutic interventions, while minimizing their impact on epigenetic modifications in normal cells. This predicament, which has been previously documented [84], underscores the importance of formulating DNMTis with tumor cell selectivity. The close relationship between demethylation and tumorigenesis highlights the importance of developing tailored TET inhibitors.

Third, the strategic integration of methylation-targeting drugs with alternative antitumor agents and methodologies warrants rigorous consideration. Monotherapeutic modalities are often associated with hurdles such as drug resistance and substantial side effects. Resolving these challenges hinges on the synergy engendered by the co-administration of methylation-targeting drugs along with therapeutic modalities, such as radiotherapy, chemotherapy, vaccines, immune checkpoint inhibitors (ICIs), oncolytic viruses, CAR-T/NK cells, and other technological approaches. Attaining a synergistic therapeutic effect coupled with the minimization of adverse effects via the judicious amalgamation of methylation-targeting drugs and other therapeutic strategies remains a formidable challenge in the future.

In conclusion, although the study of the regulation of DNA methylation in the context of immunotherapy holds immense promise, this landscape is framed by various challenges and opportunities. We anticipate advances at the exciting intersection of science and medicine in the coming years.

## Data Availability

All data generated or analyzed during this study are included in this published article.

## Abbreviations

DNA: Deoxyribonucleic acid
RNA: Ribonucleic acid
5mC: 5-Methylcytosine
TME: Tumor microenvironment
TIME: Tumor immune microenvironment
DNMT: DNA methyltransferase
TET: Ten–Eleven translocation
CTL: Cytotoxic T lymphocytes
Tregs: Regulatory T-cells
TAMs: Tumor-associated macrophages
NK: Natural killer
TGF-β: Transforming growth factor-β
DCs: Dendritic cells
ECM: Extracellular matrix
CRC: Colorectal cancer
NSCLC: Non-small cell lung cancer
NEFM: Neurofilament medium polypeptide
IFN: Interferon
MDSCs: Myeloid-derived suppressor cells
PD-L1: Programmed death-ligand 1
TNC: Tenascin-C
NPC: Nasopharyngeal carcinoma
EMT: Epithelial–mesenchymal transition
TF: Transcription factor
5AZA-C: 5-Azacytidine
DFMO: α-Difluoromethylornithine
VEGFA: Vascular endothelial growth factor A
VEGFR2: Vascular endothelial growth factor receptor 2
TSDR: Treg-specific demethylation region
KG: Ketoglutarate
IL: Interleukin
IFNG: Interferon gamma
GZMB: Granzyme B
TNF: Tumor necrosis factor
RIP1: Receptor-interacting protein kinase 1
TAM-1s: Tumor-associated macrophage type 1
TAM-2s: Tumor-associated macrophage type 2
M1: M1 macrophages
M2: M2 macrophages
CAR-T: Chimeric antigen receptor T-cell
CAR-NK: Chimeric antigen receptor natural killer cells
ICIs: Immune checkpoint inhibitors
Th2: T helper type 2
Th1: T helper type 1
CpG: Cytosine–phosphate–Guanine
CTLs: Cytotoxic T lymphocytes
IFN-γ: Interferon-gamma
PDA: Pancreatic ductal adenocarcinoma
MHC: Major histocompatibility complex
FOXP3: Forkhead box protein 3
STAT5: Signal transducer and activator of transcription 5
MeTIL: Methylation of TILs
DAC: Decitabine
ZSCAN18: Zinc finger and SCAN domain-containing protein 18
